# Beyond the brotherhood: Skoal Bandits’ role in the evolution of marketing moist smokeless tobacco pouches

**DOI:** 10.1186/s12971-017-0150-y

**Published:** 2017-12-19

**Authors:** Yogi H. Hendlin, Jessica R. Veffer, M. Jane Lewis, Pamela M. Ling

**Affiliations:** 1 Center for Tobacco Control Research and Education, University of California San Francisco, San Francisco, USA; 2 Rutgers School of Public Health, New Jersey, USA; 3 Department of Medicine, Division of General Internal Medicine, University of California San Francisco, Box 1390, 530 Parnassus Avenue, Suite 366, San Francisco, CA 94143-1390 USA

**Keywords:** Moist snuff tobacco, Tobacco industry marketing, Product innovation, Changing demographics, Redefining masculinity

## Abstract

**Background:**

Since 2006, “snus” smokeless tobacco has been sold in the U.S.. However, U.S. Smokeless Tobacco (USST) and Swedish Match developed and marketed pouched moist snuff tobacco (MST) since 1973.

**Methods:**

Analysis of previously secret tobacco documents, advertisements and trade press.

**Results:**

USST partnered with Swedish Match, forming United Scandia International to develop pouch products as part of the “Lotus Project.” Pouched MST was not commonly used, either in Sweden or the U.S. prior to the Lotus Project’s innovation in 1973. The project aimed to transform smokeless tobacco from being perceived as an “unsightly habit of old men” into a relevant, socially acceptable urban activity, targeting 15–35 year-old men. While USST’s initial pouched product “Good Luck,” never gained mainstream traction, Skoal Bandits captured significant market share after its 1983 introduction. Internal market research found that smokers generally used Skoal Bandits in smokefree environments, yet continued to smoke cigarettes in other contexts. Over time, pouch products increasingly featured increased flavor, size, nicotine strength and user imagery variation.

**Conclusions:**

Marlboro and Camel Snus advertising mirrors historical advertising for Skoal Bandits, designed to recruit new users and smokers subjected to smokefree places. Despite serious efforts, pouched MST marketing has been unable to dispel its association with traditional smokeless tobacco stereotypes as macho and rural. Public education efforts to discourage new users and dual use of MST and cigarettes should emphasize that “new” pouch products are simply repackaging “old” smokeless tobacco.

## Background

Smokeless tobacco pouch products (such as Skoal Bandits or Grizzly Pouches) and snus are subcategories of traditional moist snuff (MST): shredded, flavored, chemically-treated, fermented tobacco, normally sold in a round can. Smokeless tobacco pouch product sales more than doubled in market share in the US between 2005 and 2014, accounting for 19% of total moist snuff (MST) sales in 2014 [1]. The increase in “pouched” forms of smokeless tobacco use in the last decade reflects a general diversification of smokeless products, aiming to appeal to a broader audience than the traditional rural, white, and lower-socioeconomic male consumer [2–6], which USST referred to informally as the “Brotherhood” [7].

Tobacco companies have put significant resources into marketing and developing smokeless pouched tobacco products [8–13]. MST manufacturers have introduced over 10 new MST pouch products since 2006 (Table 1), and during that time at least four major tobacco companies introduced pouched snus products to test markets nationwide [10, 11, 14]. Domestic spending on advertising MST reached $345.4 million in 2012 [15], a 277% increase since 1998 [16]. U.S. Smokeless Tobacco (USST) President Daniel Butler pointed out during a 2007 earnings report call, that “very disproportionately, while pouches are about 5.6% of [the MST] category volume, they’ve a much higher share among new-to-category consumers” [17]. Since USST’s acquisition by Altria in 2008, multiple portion-pouch derivative products have been developed, including Skoal X-tra, Skoal Snus, Copenhagen Pouches, and Copenhagen Snus [18].

**Table 1:** Development and proliferation of pouched MST products

Pouched product brand	Parent company	Moist/Dry	Flavors	Years active
Good Luck Sak-Pak	USST	Moist	Mint	1973–1983
Skoal Bandits	USST	Moist	Straight, Mint, Wintergreen	1983-
Renegades	Pinkerton	Moist	Mint, Wintergreen	1985-
Skoal Flavor Packs	USST	Moist	Mint, Cinnamon	1993–1999
Revel	USST	Moist	Mint, Wintergreen, Cinnamon	2001–2006
Skoal Pouches (previously “Flavorflow Pouches,” “X-tra”)	USST	Moist	Rich, Crisp, Wintergreen, Mint	2008-
Skoal Dry	USST	Dry	Menthol, Regular, Cinnamon	2006–2008
Skoal Snus (replaced Skoal Dry)	USST/Altria	Dry	Mint, Smooth Mint	2011-
Grizzly Pouches	Conwood /RAI	Moist	Snuff, Straight, Mint, Wintergreen, Dark Wintergreen	2008-
Copenhagen Pouches	USST/Altria	Moist	Original, Wintergreen	2001-
Copenhagen Snus	USST/Altria	Dry	Natural, Mint, Wintergreen	Test-marketed starting 2014
Marlboro Taboka	Altria	Dry	Original, Green	Test-marketed 2006–2008
Marlboro Snus	Altria	Dry	Rich, Mild, Peppermint, Spice	2007–2013 (relaunched in 2010)
Camel Snus	RAI	Dry	Frost, Spice, Original, Robust, Winterchill	2006-
General	Swedish Match	Dry	Original, White	More heavily in the US after 2006
Catch Dry	Swedish Match	Dry	Eucalyptus, Licorice, Vanilla, Coffee	More heavily in the US after 2006

Previous research has emphasized the changing smokeless tobacco market with the introduction of Swedish-styled snus, [10] exposed USST’s “graduation” strategy to lead new users from lower to higher nicotine-delivery products, [13] and examined tobacco industry interest in smokeless tobacco within the European context [19]. This article examines tobacco industry interest in the development of pouched MST products, and the role these pouched products play in increasing the social acceptability of smokeless tobacco among different groups.

To better understand the original rationale for marketing pouched tobacco products and the factors driving their proliferation, we reviewed previously secret tobacco industry documents related to USST’s short-, mid-, and long-term planning and marketing strategies for Skoal Bandits and other pouched smokeless tobacco products. Our basic research questions included: “What factors were internally regarded as important in the development and release of Skoal Bandits and other smokeless pouch products?” and “What marketing strategies were employed during the introduction of pouch products, how did these strategies change over time, and were they successful?”

## Methods

We searched previously secret tobacco industry document archives from the University of California, San Francisco Truth (formerly Legacy) Tobacco Documents Library (https://industrydocumentslibrary.ucsf.edu/tobacco), between January 2010 and October 2010, updating searches in November and December 2015. Initial search terms included: “snus,” “Skoal Bandits,” “consumer research,” “product research,” “strategic plan,” “roll out,” and “marketing.” Initial searches produced hundreds of documents relevant to either Skoal Bandits or marketing smokeless tobacco pouch products. These searches were narrowed using more specific keywords suggested by an initial review of the first documents retrieved (e.g. “Bandits Task Force,” “ethnic research,” “Lotus Project,” “Good Luck,” “radar studies,” “college marketing”), followed by “snowball” searches using standard techniques [20]. The documents were reviewed and organized chronologically and by topic area, and summaries were written and reviewed by multiple authors. Documents were categorized using names, locations, project titles (e.g., Lotus Project), brand names, dates and Bates (reference) numbers. Outstanding question were resolved by additional searches, or via corroborating evidence from external sources, such as advertising archives like Trinkets and Trash, general internet searches, company annual reports, investors’ webcasts and trade press including Tobacco Reporter (1971–2015), Convenience Store News (2004–2015), and Smokeshop Magazine (2003–2015). Analysis was based on a final collection of 217 tobacco documents, 11 USST annual reports (1997 to 2007), 4 Altria quarterly reports (2008–2015), and 31 articles from tobacco trade press published between 1974 and 2015.

## Results

### Origins of pouch products: The good luck Sak-Pak

**Fig. 1:**
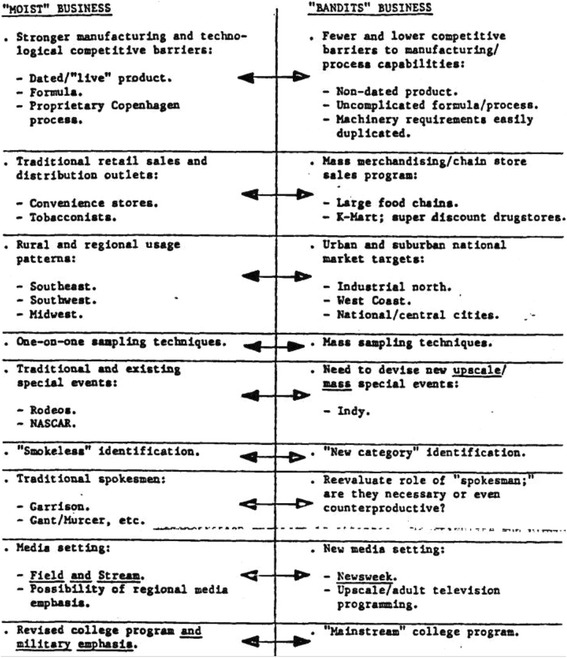
Internal USST document comparing the  Benefits and Branding of “Bandits” Pouched MST over Traditional MST [24]

The first test-marketed pouched product in the U.S., the “Good Luck Sak-Pak” grew out of the Lotus Project, a collaboration between USST and the Swedish Tobacco Company (now Swedish Match AB) starting in 1970 to develop a pouched smokeless product for Europe [21, 22]. The two companies created a joint subsidiary, United Scandia International (USI), with the goal of creating a pouched MST product that could be successful in the U.S. and internationally. USST’s executives expressed interest in new smokeless tobacco products that would counteract the socially unacceptable aspects of smokeless tobacco, such as the sensation of floating tobacco strands in one’s mouth, the messiness of spitting, and the impression that smokeless products were traditionally confined to rural and blue-collar consumers [23, 24]. USST’s Senior Vice President Stanley Beetham believed this promising product would spark a “new business,” [25] broader than the traditional MST one (Fig. 1).

The primary difference between pouched and traditional MST was seen as its main advantage: encapsulating tobacco shreds in a pouch made dipping kinesthetically easier and appear cleaner. Containing MST in tea bag-like sachets overcame what USST’s smoker focus groups identified as the top barriers deterring them from continuing traditional MST use after receiving free samples: (1) the messiness of “float” (strands of tobacco in the mouth), (2) the “lipburn” from tobacco sitting directly on the gum and lip, and (3) user anxiety over ascertaining the optimal “size of pinch” [22].

**Fig. 2:**
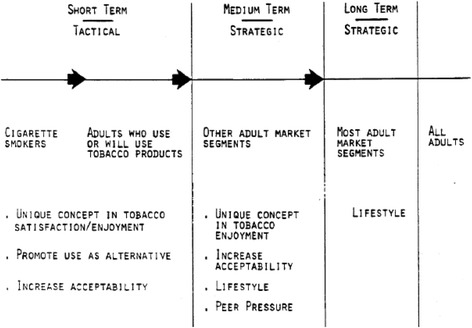
USST’s Short-, Medium-, and Long-term Strategies to market smokeless tobacco pouches [43]

When tested by moist snuff users, the portion pack was described as “hygienic, practical, easy to handle, clean, more discreet, does not flow—lies still and good in mouth” [24, 26]. USST Director of Marketing T.D. Pickett outlined different potential audiences: (1) preexisting snuff consumers, who would be attracted by the advantages of the portion pack (e.g., no loose tobacco floating in the mouth, more hygienic, convenient); (2) male smokers who would be motivated by the relatively cheaper price, notions that smokeless tobacco was healthier and “a way to avoid tar and carbon monoxide,” and accessible in situations prohibiting smoking; and (3) new “consumers not yet using tobacco products” (Fig. 2) [26]. This last group, consisting of youth and young adults, would be marketed to similarly to as the smokers, placing additional emphasis on psychological factors (peer pressure, group belongingness, popularity) [26].

USST test-marketed the Good Luck Sak-Pak between 1973 and 1983 as a key component to “change the make-up of its user base” [27]. USST’s executive officers intended to “reduce the average age of the [MST] user to the 30 to 40 year old age group from the 45 and over age group” and “increase usage by white collar workers,” a 1974 Furman Selz consulting firm report outlined [28, 29].

USST executives positioned “smokeless tobacco in a portion pack” to immediately appeal to target groups of “new users, mainly cigarette smokers, age group 15-35,” [30] while simultaneously introducing the product as “upscale” [31]. While blue collar workers often used smokeless tobacco in work environments where smoking was considered dangerous (e.g., factories) and in outside locations where spitting was not stigmatized (e.g., construction sites), such conducive opportunities to use MST did not extend to white collar workers inhabiting offices and cubicles [32]. The Good Luck Sak-Pak was intended to be an “entirely new product aimed at new consumers,” which could transform the public image—and social acceptability—of smokeless tobacco [30].

When Good Luck Sak-Pak test-marketing revealed that “over 10% of the people trying it [were] non-smokers”—a much higher recruitment rate for never-smokers than regular snuff—USST executives grasped pouched MST’s potential to expand the smokeless market [28]. Marketing in the 1970s, USST distributed free samples to first time users, instructing seasoned company representatives (reps) to walk novices through the mechanics of placing the pouch between the upper lip and gum, the length of time the pouch should stay before removal, the “issue of salivation,” and above all, reps were to encourage users not to give up should their first experience(s) be less than pleasant [33].

Selling the *idea* of pouched smokeless as a novel category became as important as selling the product itself [25, 34]. USST Division Manager R.R. Marconi’s 1973 memo to R.*L. Rossi*, Director of Sales, noted that the success of the Lotus Project depended on “education” programs. The challenge was how to “educate” the public about pouches’ distinction to steer the image of smokeless tobacco away from stereotypes of “an old man sitting in a rocking chair trying to hit a cuspidor ten feet away” [26, 34].

Along with Good Luck, a sweet flavored low nicotine non-pouched MST product called “Happy Days” also emerged from the Lotus Project. These products formed USST’s “Starter Product” category, regarded as a “*transient* market segment” as users would soon graduate to higher nicotine products upon habituation [35]. Both products were conceived as stepping stones to USST’s more established and higher nicotine Skoal and Copenhagen; but due to Good Luck and Happy Days’ insufficient nicotine delivery and hippy imagery, these starter products were soon displaced by Skoal Bandits as the flagship starter product [11, 23, 35–38].

### The Skoal bandits brand extension

USST sold Good Luck in limited test markets until it was discontinued and replaced by the launch of Skoal Bandits in 1983 (Good Luck continued to be sold internationally until 1990) [39]. Linking the easy-initiation pouched product with the longstanding Skoal brand—second in brand recognition only to Marlboro among all U.S. tobacco brands at the time [40]—overcame the obscurity and perception of the unfortunately-named Good Luck Sak-Pak as potentially a “sissy product,” a perception that would follow pouched MST for decades through its many iterations [35, 36]. USST essentially took Good Luck and rebranded it as Skoal Bandits according to the more masculine, nationally-distributed, and broadly recognized Skoal brand name.

The name “Bandits” given to the rebranded pouched MST product coincided with USST’s eponymous sponsored race car, the “Skoal Bandit;” the NASCAR racer strategically debuted a year before the pouched product launch, replete with branded racing gear bearing the same logo as would grace the product [41, 42]. By November 1983, USST had a 90 % share of the moist smokeless tobacco market, and Bandits built on Skoal’s success through a combination of promotional strategies which included auto racing, rodeo and skiing events [43, 44]. By May 1984 an *Advertising Age* survey found that due to USST’s unprecedented marketing campaign, Skoal Bandits (separate from Skoal) already was the eighth most recognized tobacco brand overall, less than a year after its roll-out [40].

Part of USST’s impetus for developing Skoal Bandits was in response to competition in the starter MST category by other tobacco companies. In 1979 Conwood Co. (now RJ Reynolds’ American Snuff Company) introduced Hawken, a sweet tasting, manageable “easy-balling” (bolus-forming) cherry MST brand popular with youth [45]. Conwood’s strategy was to initiate starters with Hawken and then enroll them into its higher-nicotine Kodiak brand, [45] potentially disrupting USST’s graduation funnel moving Good Luck/Bandits initiators to its stronger unpouched Skoal and Copenhagen brands. To compete, Bandits’ 1983 public launch became USST’s biggest advertising campaign expenditure to date [45, 46].

To support marketing for this line extension of its most popular Skoal brand, USST created the Skoal Bandits Task Force, comprised of high-ranking company executives. The Task Force planned to focus MST marketing for the first time on a broader, mainstream target audience [47, 48]. While Skoal Bandits was test-marketed in eight Southeastern cities in 1982, [49] it was officially launched as a national product with a press conference in New York City in August of 1983, with an unprecedented $2 million advertising campaign for the New York City market alone [50]. Ex-Dallas Cowboy Walt Garrison, a Skoal paid spokesman, explained, “If you can sell New Yorkers on snuff, it’s a piece of cake everywhere else” [51]. USST’s investment in advertising was expected to yield high returns: pouched products also increased profit margins, as Bandits sold for the same price as regular Skoal or Copenhagen MST but only contained one-fourth as much actual tobacco in the can [52]. During the 1980’s, Bandits was USST’s highest gross profit margin product [52].

The Skoal Bandits pouch products aimed to recruit young never-users of tobacco as well as appeal to smokers interested in quitting [48]. The Bandits Task Force discussed market segmentation for the first time in a 1983 meeting, and identified smokers as the largest cluster of Bandits consumers in the short term [44]. USST’s medium and long-term strategies for Bandits included a wide variety of new users (Fig. 2) [44].

### Short term goal: Appeal to smokers

Advertisements framed pouched MST as sophisticated, easy-to-use, and socially acceptable [48, 53–55]. USST CEO Bantle told reporters as early as 1983, “We see our product concept taking advantage of the rising tide of dissatisfaction among smokers with the social inconveniences of cigarettes” [35]. The earliest advertisements for Skoal Bandits emphasized use as an alternative to smoking, particularly in smokefree environments. These ads suggested substituting Bandits for cigarettes, applying the slogan, “take a pouch instead of a puff”; though this slogan was discontinued in May 1984 after USST paid $25,000 in legal costs to settle New York State Attorney General Robert Abrams’ suit claiming that USST’s advertising copy implied Bandits constituted a safe alternative to cigarette smoking [56–58].

USST’s internal market research showed Bandits’ high acceptance among smokers, as both a cigarette substitute and for dual intermittent use along with cigarettes. Shortly after Bandits’ introduction, USST conducted a survey of 223 self-identified smokers who had tried Skoal Bandits, including a mix of smokeless users and non-tobacco users. Of the smokers, 53.9% said they would completely substitute Bandits instead of smoking cigarettes, 42.6% would use Bandits in addition to smoking cigarettes, and only 3.6% would not use Bandits again [59]. A 1984 National Survey of Moist Snuff Users conducted for USST found that “Skoal Bandits was more likely than any other smokeless tobacco product to be used in conjunction with cigarette smoking” [39].

**Fig. 3:**
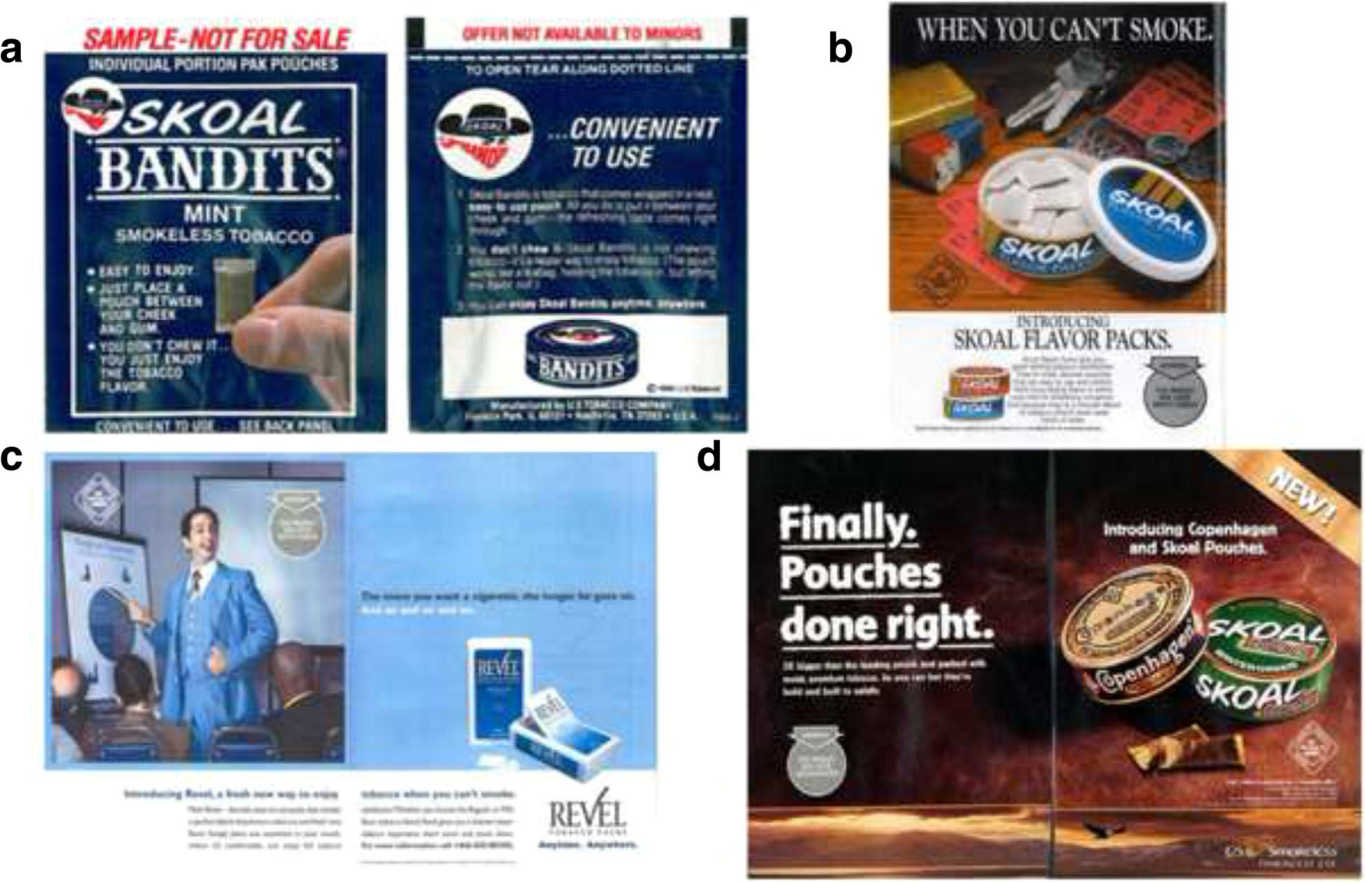
Pouched Product Evolution. **a** Skoal Bandits Mint (1983) easy-to-use directions enabled self-sampling. By having the tobacco contained in a pouch, USST could send these products out via mail and give them out at events, without the onerous one-to-one demonstrations required to initiate unpouched MST use. **b** 1993 Skoal Flavor Packs played off the rise of smokefree indoor air laws, highlighting their ability to deliver nicotine (“satisfaction”) and their status as “small, discreet pouches.” **c** Revel’s (2002) pastel colors, clean look, and Tic-Tac™ shaped box targeted white-collar workers, distancing this pouched MST product from the rough outlaw image Bandits had cultivated. This ad shows a woman, an African-American man, and a balding white man in a white-collar business environment as composing Revel’s target audience. **d** The opposite of Revel, Skoal and Copenhagen Pouches (2002), “3× bigger” than Bandits, are part of the emerging market for high-nicotine pouched products, like Grizzly’s. MST users no longer are limited to lower-nicotine pouched products before graduating to loose snuff, but instead pouched MST aims to continue tobacco addiction without the user ever having to use unpouched MST. This ad appeared, among other places, in the young male-targeted magazine Popular Mechanics

Over the years, USST introduced more pouch products, marketed explicitly as situational substitutes for smokers under the Skoal brand. USST began selling the emulative Skoal Flavor Packs in 1993 to “appeal to smokers who do not currently use smokeless tobacco” as a situational substitute for smoking [60]. Skoal Flavor Packs advertising features a pack of cigarettes and a lighter placed alongside car keys, ballgame ticket stubs, and a tin of Skoal Flavor Packs with the caption, “When you can’t smoke” (Fig. 3b), reminiscent of Wrigley’s spearmint gum’s “When I can’t smoke, I enjoy pure chewing satisfaction” commercials popular in the early 1990’s [61, 62].

In the early 2000s, new pouched products appeared from USST, Conwood, and Pinkerton, the three largest smokeless tobacco manufacturers (Table 1). This wave of pouched products introduced for the first time high-nicotine pouched MST, appealing to already-established smokeless users and heavy smokers. In 2004, USST introduced another spin-off, Skoal Pouches, advertised as “3× larger than Skoal Bandits,” marketing it as a “solution” for smokers in smoke-free venues (Fig. 3d).While Skoal Pouches delivered substantially more nicotine than Bandits, USST’s advertising of the product as convenient and discreet retained brand positioning. USST also introduced a new pouched MST product, Revel, in 2001, which emphasized use in smoke-free environments but featured less rugged traditional brand imagery, aimed at a more diverse and metropolitan audience (Fig. 3c) [63].

In 2006 the leading U.S. cigarette companies, Philip Morris ([PM] now Altria) and Reynolds American (RAI), entered the smokeless market with their Taboka and Camel Snus products, respectively [64]. Taboka, PM’s first MST pouch product, was marketed to “fill a niche for smokers seeking a palatable option for quitting” [64]. RAI’s Camel Snus emulated Swedish snus and employed the exoticism of refined European culture and Swedish design to sell their pouched product as new and different. In response to these new products, USST replaced Revel with Skoal Dry pouches, which, similar to Camel Snus, did not require spitting (Fig. 3).

Smokeless tobacco companies faced several hurdles to getting smokers to switch to smokeless or to supplement smoking with MST. First, nicotine delivery from smokeless tobacco is slower than cigarettes [65]. Second, most smokers perceived smokeless tobacco as unsophisticated [66]. Portion pouches, and later snus, advertising issued constant reminders that these perennially “new” products were “not dip” [6, 67].

### Longer term goal: Pouch products as a way to expand the market

Bandits’ brand recognition and ease-of-use opened new marketing opportunities for USST, including for the first time, mass sampling (free sample mailing and distributing at events) and a broader array of media outlets, distribution channels, and user groups [47]. Rather than the niche marketing and tedious one-on-one sampling previously employed to teach people how to use traditional MST, [44] sample packs of Bandits came with step-by-step instructions on their wrapper explaining how to use them (Fig. 3a). Mass distribution of free samples dramatically increased the scale of Bandits promotions, while advertising emphasized two staple themes (1) appealing to smokers and (2) recruiting new users among groups not already using MST. USST Director of Sales Ralph L. Rossi wrote, “I would think that we would want to go after the younger smoker, especially around the college campuses” [34]. This is what USST did [68].

Marketing plans in the 1980’s and 90’s emphasized attracting new users through lifestyle marketing aimed at groups like college students and ethnic markets [35, 69, 70]. Recommendations from the Bandits Task Force included implementing an educational (how the product was different) and instructional (how to use it) component into advertising and expand into “micro markets” such as African-American, Hispanic, college, and urban white-collar populations [26, 70].

Steve Africk, the College Program Coordinator, noted that the college market would solidify USST’s future in the smokeless market, especially amongst “the 18-34 age group[…] [and the] more than 12 million college students throughout the country” [71]. To reinforce advertising, USST’s College Marketing Department recruited and paid college-going representatives to form male smokeless tobacco clubs on campus, distribute samples, collaborate with retail vendors, organize special events, and conduct on-campus promotions [72]. Mass sampling events took place in conjunction with other campus-sanctioned activities, and at locations where students gathered (e.g., spring break parties and vacation locales) [70, 73]. College representatives were instructed to educate samplers and future users about how to use Skoal Bandits, dispel prejudices against smokeless tobacco, and to do everything in their power to make Bandits look cool [74].

The USST College Marketing program newsletter *Smokeless Signals* included glowing reports from college representatives about their experiences distributing free samples. Student rep Joseph Augustus shared in *Smoke Signals*, “Being in the Northeast most people are not very familiar with smokeless tobacco products. A sampling program such as this is a great eye opener. Many of the students who have vowed never to use tobacco are taking a dip on a semi-regular basis after realizing the pleasure” [42]. The college marketing program provided not only a method to disseminate product, but valuable feedback for USST to determine what products enticed young adults.

While smokeless tobacco was perceived in the 1970s as something used by older, rural, mostly Caucasian men, initial success in converting new users and lowering the age of the average user led USST to explore Skoal Bandits as a means to appeal to groups previously not considered viable for smokeless tobacco marketing. With the advent of Bandits, African-American and Hispanic populations became “relatively untapped” markets for smokeless tobacco revenue [75]. USST’s prime interest in attracting African-American and Hispanic users derived from the high proportion of under 35 men of color, based on statistics predicting these populations would increase in coming decades [76–78]. USST’s marketing consultants drew up plans for converting African-Americans and Hispanic men, [69, 78] who USST viewed as already primed for smokeless tobacco advertising by virtue of inhabiting “highly individualistic and macho” cultures [75].

Surprisingly, USST also considered marketing Bandits to women [70, 76, 79]. Consultants and executives recognized what they believed to be pouched products’ “[p]otential appeal to both sexes,” “multi-lifestyle” and multi-ethnic attraction, cultivating consumers living—or at least aspiring to live—a “more upscale/urban lifestyle” [44]. However, neither Skoal Bandits nor other pouched MST caught on significantly in these nontraditional smokeless tobacco demographics, for reasons discussed below [5].

## Discussion

Selling MST in pouched form became an important strategy for lowering barriers to initiation among new smokeless tobacco users, whether for preexisting smokers or new tobacco users [13, 80]. Because of its contained ease of use, lower free nicotine content, [11] and brand positioning as a different product from other forms of MST, pouched product sales have become a major segment of the current smokeless tobacco market.

Part of the growth of smokeless tobacco can be attributed to pouch products’ ability to attract a broader consumer base than traditional MST [81]. The cigarette companies’ entry into smokeless marketing though acquiring smokeless tobacco companies, including Reynolds American buying Conwood for $3.5 billion in 2006 and Altria buying USST for $10.4 billion in 2009, was followed by the introduction of Camel Snus, Taboka, and Marlboro Snus [81]. In its acquisition of Conwood smokeless tobacco company, RAI acquired the discount brand, Grizzly, and introduced Grizzly pouches in 2008. The Grizzly brand now has the largest smokeless tobacco market share [5]. The increase in consumer awareness of RAI and PM’s snus products (and their extensive advertising) increased overall pouched MST awareness and usage [17, 82]. Multiple convenience store and tobacco industry magazines reported in 2010 that “portion packs [including the new spitless category of portion pack, snus] are the fastest growing segment in the smokeless tobacco category” [64, 83, 84]. As of 2015 every major U.S. tobacco company portfolio included multiple pouched products [11]. This shift in the American tobacco industry’s product emphasis toward more approachable pouched smokeless products, including snus, signals a strategic landmark, transforming the niche product of smokeless tobacco into a convenient and discreet form with broad marketing possibilities.

### The success of the lotus project

Pouched products have grown into a lynchpin MST starter product, not just for USST, but all MST manufacturers. A much wider variety of pouched products are now available. While at the inception of the Lotus Project, pouch products' users were expected to graduate to the higher nicotine brands of loose MST, Skoal and Copenhagen, [13, 33] over time pouch product variants of higher nicotine MST have also been introduced, including large size pouches of Skoal, Grizzly, and Copenhagen (Table 1). The average free nicotine in pouched MST has also dramatically increased, [11, 65] demonstrating the creation of a parallel graduation process of exclusively pouched MST products that range from low to high nicotine levels. The repeated introduction and promotion of pouched products in their myriad forms may have contributed to increasing the current smokeless tobacco prevalence to 3.0%, an order of magnitude greater than the *Healthy People 2020* objective [85].

Pouched products may have also contributed to major shifts in the demographic profiles of smokeless tobacco users. In 1970, 12.7% of men sixty-five or older used smokeless tobacco; but by 1991, the period after portion pouches were introduced, only 5.6% of older men used smokeless tobacco. During the same period, young male adult rates of smokeless tobacco use rose from 2.2 to 8.4%, resulting in a net increase in total smokeless tobacco prevalence [86]. The CDC’s Michael Eriksen testified in 1994, “[s]ince 1970, the epidemiology of smokeless tobacco has virtually reversed itself. No longer is smokeless tobacco a habit of older men; the highest use now occurs among young white men who increasingly choose moist snuff products” [86]. This trend over the past three decades suggests the main goal USST had initially set out for the Lotus Project was achieved [34].

### The tenacity of the brotherhood

While the Lotus Project strategies may have contributed to the increasingly younger age of the average pouch user, [34, 80, 87] smokeless tobacco pouches were also used to attract customers beyond the “Brotherhood,” the rural and rugged white male core users of smokeless products characterized by Skoal advertising. Pouched MST products were also aimed at women, minorities, and urban white-collar workers. Yet, MST continues to have limited appeal outside of its traditional rural male demographic.

One possible explanation for the lackluster response of minorities and women to MST could be inconsistencies in marketing; namely, the tension between the putative inclusiveness of neat pouches and the macho exclusiveness of the Skoal brand. On the one hand, Skoal Bandits advertising aimed to reconfirm that “Skoal is part of an individualistic, masculine lifestyle,” buttressing USST’s existing consumer base [36]. Skoal products continued to be marketed with this image of a tough, outdoor/hunter, patriotic, white, masculine clan of users, such as the recent Skoal advertisements in Playboy magazine (including an entire Skoal-branded issue of Playboy) [7]. Skoal-using Playboy readers were named members of the “Skoal Brotherhood,” and encouraged to visit the USST website by the same name (www.SkoalBrotherhood.com). Admittedly, the majority of these tough-guy advertisements were for loose Skoal MST—not the pouched variety. However, one ad in the magazine for oversized Skoal Pouches reads: “There are times you want a straight dip that’s fast and easy. Even if you'll never admit it” [88]. Such ad copy verbalizes and so diffuses the Brotherhood of dippers’ contempt for pouched MST users, who might otherwise be regarded as lightweights or phonies.

On the other hand, USST recognized Bandits and subsequent niche pouched products such as Revel as an unparalleled opportunity to broaden smokeless tobacco’s image to embrace female and minority user groups [44, 70, 79]. This subversion of the Skoal Brotherhood’s dominant MST image by creating pouched products to attract those potential smokeless users not resonating with the traditional imagery was a pillar of the Lotus Project. Making smokeless tobacco mainstream and mass-marketable was their goal, and if increased usage is the benchmark of success, this strategy has been successful.

This split between the profiles of traditional MST users and pouched MST users may have hampered the success of USST’s plan to differentiate pouches from MST. The cognitive dissonance within the Skoal brand, which includes both pouched and loose products, potentially drives traditional users away from a watered-down Skoal brand, while the lingering existence of tough male chauvinist imagery also blocks potential smokeless users from taking up pouched products.

### Pouched products are still smokeless tobacco

The tension between delivering a “new” product while also retaining the strength of an established brand recurs throughout tobacco product marketing [81, 89]. Pouched MST and pouched snus first came to market in 1973 in the U.S. and Sweden, respectively, resulting from the Lotus Project. Yet, pouched products were repeatedly and effectively re-introduced in the US as “new” to convince fresh generations of users that pouched MST confers alternative identity configurations from the less attractive ones traditionally associated with smokeless tobacco. Lingering anxiety about needing to educate the public on this “relatively new” “spit-free small pouch category” persisted even in 2007, driving USST president Murray to declare that USST “welcome[d] the competition” of RAI and Altria’s snus products to expand the effort USST had expended explaining to the public how pouched snuff is different from dip [17]. However, marketers have largely failed to move broader society away from longstanding perceptions that smokeless tobacco use is a messy, low-status product [67].

These data suggest that public health practitioners concerned about the introduction of “new” tobacco products that appeal to youth should encourage consumers to question whether these products are actually “new and improved,” and instead recognize them as repackaged tobacco products rejected by similar consumers in the past. Increasing warning label size and including graphic warnings would reduce youth initiation to smokeless tobacco, [90] accenting the fact that pouched products are still tobacco products, even if branding for certain products attempts to distance it from traditional MST. For existing smokers, cessation treatments must emphasize that attempting to quit by switching to smokeless tobacco is not effective, [91] and this strategy still exposes users to known carcinogens.

## Conclusions

Historical analysis of the U.S. introduction of pouched smokeless products provides insight into how pouched products became the current fastest growing segment of the U.S. smokeless tobacco market. Initially the product’s physical ease of use, low nicotine levels and appealing flavors allowed it to be mass marketed to beginners and obviate painstaking one-on-one sampling. The introduction of multiple variants of pouch products with an increased range of nicotine strength, coupled with the claims that the products are “new,” led to increases in both pouched MST and sales of snus products in the U.S. A major barrier to widespread adoption has been the difficulty of convincing the public that MST pouches were actually “new” and different, as well as dissonance between MST’s traditional masculine rugged advertising and the attempts to position the product for minorities or women. These factors are likely to impact the introduction and promotion of novel tobacco products in future years. Due to similar effects on youth initiation, regulatory policies to decrease youth tobacco initiation (such as restricting characterizing flavors) could be fruitfully applied to pouch products.

## Abbreviations

CDC: Centers for Disease Control
MST: Moist snuff tobacco
PM: Philip Morris
RAI: Reynolds American International
USST: United States Smokeless Tobacco Company
